# KIT-Negative Systemic Mastocytosis Associated With Acute Myeloid Leukemia

**DOI:** 10.1155/crom/3563591

**Published:** 2025-09-06

**Authors:** Kabeer Ali, Tasnuva Rashid, Jennifer Miatech, Abhinav Karan, Zachary Chandler, Gerardo Diaz Garcia, W. J. R. Quan

**Affiliations:** ^1^Department of Medicine, University of Florida College of Medicine: Jacksonville, Jacksonville, Florida, USA; ^2^Department of Pathology, University of Florida College of Medicine: Jacksonville, Jacksonville, Florida, USA; ^3^Department of Medicine, Division of Hematology and Oncology, University of Florida College of Medicine: Jacksonville, Jacksonville, Florida, USA

**Keywords:** KIT mutation, mast cell, SM-AHN, systemic mastocytosis, systemic mastocytosis associated with hematologic non-MC lineage disease

## Abstract

Systemic mastocytosis (SM) is a rare blood disorder characterized by the clonal proliferation of mast cells in tissues. Mast cells release various vasoactive mediators, including histamine, leukotrienes, prostaglandins, platelet-activating factors, and cytokines such as tumor necrosis factor. Clinical manifestations can range from mild itching to severe distributive shock. In some rare cases, mastocytosis is associated with other blood disorders, such as systemic mastocytosis with associated hematologic neoplasm (SM-AHN). Almost all cases of SM exhibit a KIT point mutation. We report a rare case of KIT-negative SM associated with acute myeloid leukemia. Historically, AML has been associated with a poor prognosis, and further research is needed to understand the prognosis of SM associated with AML. In this particular case, the patient underwent induction chemotherapy with azacitidine and venetoclax, and a follow-up bone marrow biopsy showed a reduction in mastocytosis without complete hematologic recovery. The authors aim to present this case as an example of the complex nature of SM and its diverse clinical presentations.

## 1. Introduction

Systemic mastocytosis (SM) is a rare disorder characterized by clonal expansion and proliferation of neoplastic mast cells (MCs) with subsequent accumulation in various tissues and organ systems. Depending on the extent of organ involvement, the spectrum of this hematologic neoplasm can range from isolated cutaneous mastocytosis (CM) to advanced SM [1]. The revised 2022 World Health Organization (WHO) classification of hematopoietic and lymphoid tumors classified SM as a separate central disease entity [2]. Based on the histologic findings and organ-specific damage, SM is classified into indolent SM (ISM), smoldering SM (SSM), SM with associated hematologic neoplasm (SM-AHN), aggressive SM (ASM), and mast cell leukemia (MCL) [1].

The clinicopathologic features, clinical course, and prognosis vary profoundly among the various subtypes of mastocytosis. Mastocytosis is generally considered a rare disease with no precise data on incidence or prevalence. However, a study by Brockow estimated the prevalence of 10 cases per 100,000 [3]. Mastocytosis is considered a nonhereditary somatic disease, with most cases presenting with a KIT point mutation in D816V [1]. Other somatic mutations associated with drug resistance and disease progression include TET2, ASXL1, SRSF2, CBL, RUNX1, and RAS [4]. Familial disease is rare and accounts for approximately 1%–2% of all cases [5].

The prognosis of advanced variant SM is usually poor, with an overall survival of less than 12 months [1]. SM-AHN is generally associated with myeloid neoplasms. However, there is a lack of data on SM and AML specifically. Here, we report a rare case of a 63-year-old male who was diagnosed with concurrent KIT-negative SM on a background of acute myeloid leukemia (AML).

## 2. Case Presentation

**Figure 1:**
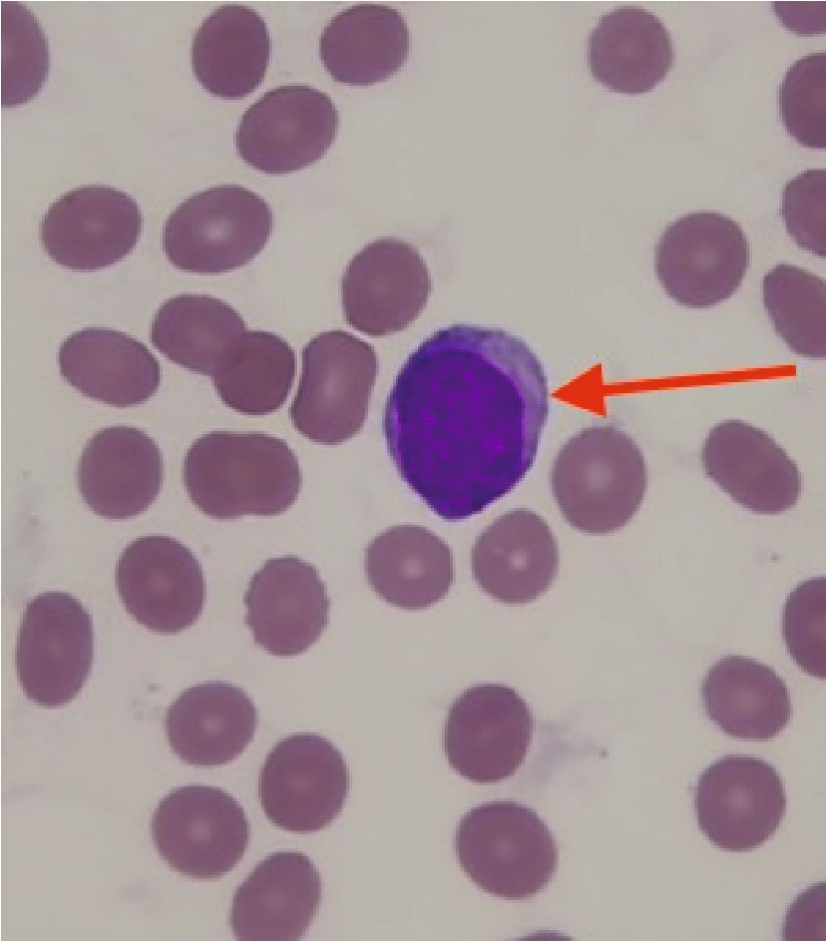
A myeloblast on the patient's peripheral blood smear.

**Figure 2:**
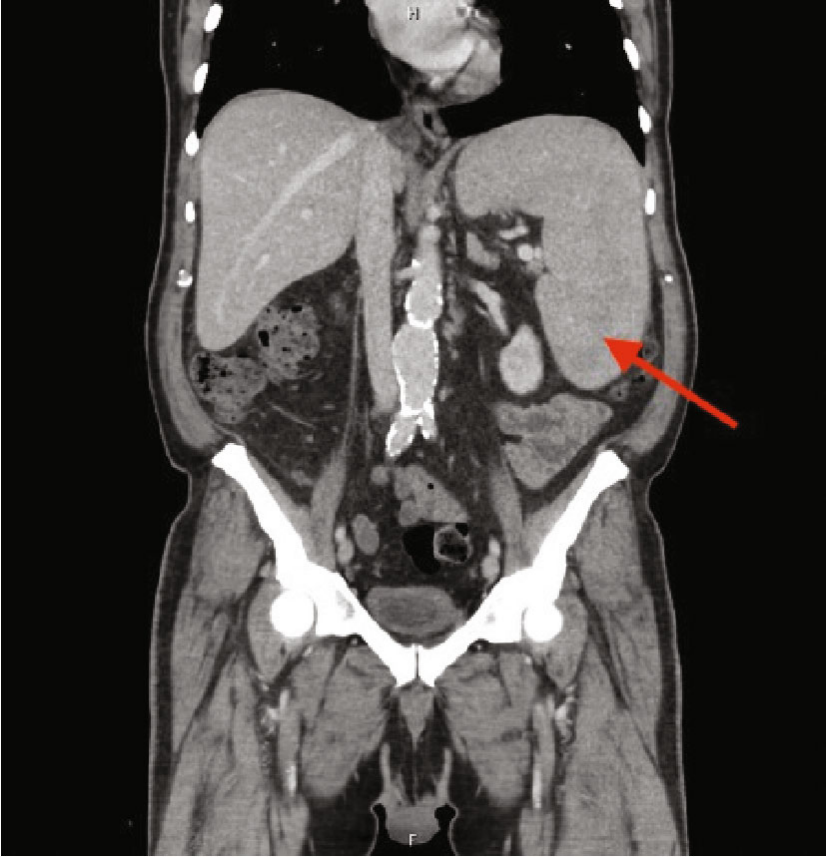
Splenomegaly out of proportion to the patient's hepatocellular disease.

A 64-year-old Caucasian male with a past medical history of coronary artery disease, chronic obstructive pulmonary disease, alcohol misuse, and current tobacco use was hospitalized for left orbital cellulitis. On admission, he was noted to have pancytopenia, with a white blood cell count (WBC) of 2.79 × 109/L, hemoglobin of 7.3 g/dL, and platelet count of 37 × 109/L. On the peripheral smear evaluation, myeloblasts, basophilia, metamyelocytes, and promyelocytes were identified (Figure 1). A computed tomography scan of his abdomen and pelvis showed splenomegaly at 18.8 cm in the craniocaudal dimension (Figure 2). Peripheral blood flow cytometry showed 5% myeloblasts (CD34/CD117).

**Figure 3:**
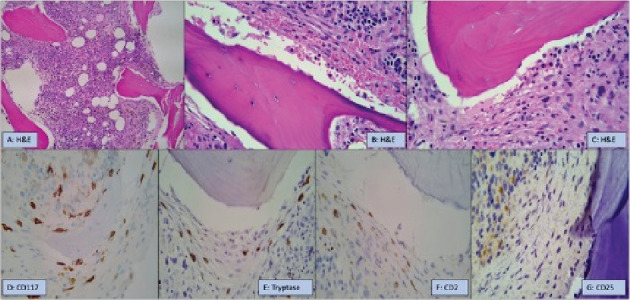
Bone marrow biopsy H&E and immunohistochemistry (IHC): (a–c) Paratrabecular clusters of spindle-shaped MCs with fibrosis compatible with systemic mastocytosis. (d, e) IHC for CD117 and tryptase highlights the MC clusters. (f) IHC for CD2 is positive in a subset of spindle cells. (g) No expression of CD25 by IHC.

Bone marrow aspirate and biopsy showed non-M3 AML involving 25 percent of the marrow cells. Notably, there were several paratrabecular clusters of spindle-shaped MCs (> 15 MCs in aggregate on CD117 stain) accompanied by fibrosis, which was compatible with SM (Figure 3). Immunochemical staining for tryptase highlighted MC clusters. Subsets of the spindle cells were CD2+, and there was no expression of CD25 by immunohistochemistry. Molecular markers were significant for detecting ASXL1 G646Wfs∗12, ASLX1 R6393∗, and PTPN11 A72V. No alterations were detected in the FLT3, IDH1, IDH2, and NPM1 genes. Cytogenetic testing revealed a complex karyotype 46, XY,7, der(11)t(7;11)(q22;q21),+mar [10]/46, idem, add(6)(q23), add(10)(q24)[cp10]. Notably, serum tryptase was measured to be 12 *μ*g/L, and KIT mutation testing on the patient's bone marrow was not detected by the Sanger sequencing method which analyzed for possible mutations in KIT Exons 8, 9, 11, 13, and 17.

The decision was made to pursue induction chemotherapy for AML with azacitidine 75 mg/m2 subcutaneously Days 1–7 and venetoclax as a dose ramp-up to 400 mg orally due to his age and multiple comorbidities. His hemodynamic status was closely monitored in-patient during this phase of treatment, which the patient tolerated reasonably well. His re-evaluation bone marrow biopsy 28 days postinduction showed a partial response, with 6% blasts by morphology, CD34+/CD117+ myeloblasts, and 2% monocytic cells without a significant change in cytogenetics. There was also a reduction in the level of mastocytosis, with only one of the clusters showing 15 MCs. He did not have complete hematologic recovery and continued on chemotherapy with a goal of disease control and palliative intent.

The patient eventually achieved a remission with incomplete hematologic recovery (CRi) following treatment with azacitidine and venetoclax. Bone marrow evaluation at that time revealed 3% myeloblasts and persistent clusters of MCs, with up to 15 cells noted. A subset of these spindle-shaped MCs expressed CD2; however, CD25 expression was absent by immunohistochemistry. Cytogenetic analysis demonstrated a persistently abnormal karyotype with interval evolution, including newly acquired abnormalities involving the long arms of chromosomes 6 and 10, which became the predominant clone.

Allogeneic hematopoietic stem cell transplantation was considered but ultimately not pursued due to patient preference and the presence of multiple comorbidities. The patient completed four cycles of azacitidine and venetoclax, with multiple dose reductions necessitated by persistent neutropenia. Following the fourth cycle, repeat bone marrow evaluation demonstrated refractory disease with 8%–10% myeloblasts by morphology and flow cytometry. Tryptase staining revealed a cluster of more than 15 spindle-shaped MCs; however, there was no overt expression of CD2 or CD25 by immunohistochemistry. Cytogenetic abnormalities remained similar to those in the prior study. Molecular testing was not performed due to limitations at the reference laboratory. In light of disease progression, treatment was switched to decitabine. Shortly thereafter, the patient presented to the hospital with pneumonia and developed septic shock, ultimately resulting in death.

## 3. Discussion

SM is a rare neoplastic hematologic process characterized by the abnormal accumulation of MCs in various organs and tissues throughout the body, including skin, bone marrow, lymph nodes, spleen, liver, and gastrointestinal tract. Bone marrow and bone involvement are present in around 90% of SM cases [6]. MCs are immune cells derived directly from hematopoietic progenitor cells that contribute to the body's response to inflammation and allergic reactions [7]. The MCs contain granules rich in proteases and vasoactive and proinflammatory mediators. Additionally, MCs contain high-affinity Immunoglobulin E (IgE) binding sites [7]. The uncommitted and MC-committed stem cells express MC-related receptor kinase KIT. The bonding of stem cell factor (SCF) to KIT initiates the development of MCs. This suggests that patients who do not express KIT, such as the patient in this case, may experience less mastocytosis and have a better prognosis. This is evidenced by other data showing that mutations in KIT D816/core-binding factor (CBF)–associated AML conferred a poor prognosis [8]. In patients with mastocytosis, SCF-independent differentiation of mastocytosis is observed. Activation of KIT on binding to SCF ligand triggers autophosphorylation and dimerization of KIT [9]. Activated KIT signals downstream protein kinase pathways, induced by cell proliferation and survival. Everyday stimuli for MC degranulation include IgE-mediated (allergens) and non-IgE-mediated (medications, hormones, exercise, cytokines, and other stimuli) mechanisms. Activated MCs release histamine, proteases, tryptase, tumor necrosis factor (TNF), phospholipase, and cytokines [10]. The most affected sites include skin and bone marrow. A 2016 survey of 460 patients in Italy highlighted the varying presentations, which can range from benign pruritus to life-threatening distributive shock [11]. Interestingly, this patient did not manifest with unprovoked dermatologic symptoms. However, he did experience “red man syndrome” during infusion of vancomycin, possibly related to underlying mastocytosis.

Due to the rarity and protean nature of the disease, a high index of suspicion is required for early diagnosis of mastocytosis. Only a few cases of KIT-negative SM have been reported in the literature. Specifically, a multicenter retrospective study identified four patients out of 40 tested who exhibited the wild-type form of the KIT gene, indicating the absence of KIT mutations [12]. This rarity complicates the diagnostic process, as the KIT mutations, particularly the D816V mutation, are present in over 80% of SM cases [13].

The diagnosis of SM, as well as the staging of the disease, requires integrated clinical, histopathologic, radiologic, laboratory, molecular, and cytologic testing [1]. The 2022 WHO diagnostic criteria for SM require the presence of the major criterion and at least one minor criterion or three or more minor criteria [14]. Major criteria include multifocal dense MC infiltrate (≥ 15 MC aggregates) detected in bone marrow or extracutaneous organs [14]. Minor criteria include (i) > 25% MCs in the bone marrow that are spindled with abnormal or atypical morphological features of MCs; (ii) detection of KIT mutation; (iii) expression of one or more MC markers: CD25, CD2, and CD30; and (iv) persistent serum total tryptase > 20 ng/mL [14, 15]. In addition, evaluation for organ dysfunction includes B and C findings, which can indicate the detection of abnormal blood count, hepatomegaly, splenomegaly, and osteolytic skeletal lesions [16]. Studies have shown that patients with advanced SM, age > 60 years, Hb < 10 g/dL, transfusion dependence, platelet < 150 × 109/L, serum albumin < 3.5 g/dL, and mutation in ASXL1 were each associated with inferior survival in multivariate analysis [17]. Although *ASXL1* mutations have been associated with increased sensitivity to venetoclax, *PTPN11* mutations may confer resistance by upregulating antiapoptotic proteins such as MCL-1 and BCL-xL, providing a potential mechanism of therapeutic resistance [18, 19]. Among patients with SM-AHN, systemic mastocytosis with associated myeloproliferative neoplasm (SM-MPN) has a significantly longer median survival than patients with systemic mastocytosis with associated chronic myelomonocytic leukemia (SM-CMML), SM-MDS, and SM-AML, which have poor prognoses [20]. ASM has an overall survival of 40 months, SM with AHN has an overall median survival of 20 months, and MC leukemia has an average survival of 24 months [13]. ISM can progress to a more aggressive form in 3% of cases [13].

The microscopic appearance of MCs includes light blue cytoplasm with granules and a central nucleus with a fried egg-like appearance with one to few prominent nucleoli [21]. Immunohistochemical stains are positive for CD117, MC tryptase, and CD25 [22]. Positive flow cytometry markers include CD117, CD25, and CD2 [22]. CD34 may highlight blast cells in cases of SM-AHN [1]. CD30 is identified in varying frequencies in early and advanced forms of SM [23, 24]. Our case's initial bone marrow biopsy demonstrated 90% cellularity and 30% CD34-positive myeloblasts. Two paratrabecular fibrotic areas with clusters of > 15 spindle-shaped MCs were seen. These clusters were positive for CD117 and tryptase, with a subset of cells positive for CD2. No overt CD25 expression was identified. He was diagnosed with SM with concurrent AML.

The majority of AHN cases are myeloid neoplasms, including CMML, AML, JAK2 mutated MPN, and MPN/MDS. Most of these cases were resistant to traditional chemotherapies with overall poor outcomes. The absence of KIT mutations necessitates a thorough evaluation for other genetic abnormalities, such as mutations in ASXL1, RUNX1, and SRSF2, which are known to impact prognosis and survival in SM-AML adversely [25]. A retrospective study of 28 cases of AML in patients with SM highlighted that blasts associated with AML could be distinguished from MC by standard morphology [26]. Similarly to our case, these patients did not express a significant tryptase elevation. The incidence of SM associated with AML needs to be better established due to the rarity of this disease. However, its incidence is increased compared to SM cases associated with preleukemic conditions such as MDS. A case series looks specifically at SM-associated at (8; 21) AML, which was not the karyotype detected in our patient, highlighting the rarity of this case [27].

A distinctive aspect of this case is the absence of typical diagnostic criteria found in SM, notably the mutation in the KIT gene. KIT is a proto-oncogene receptor tyrosine kinase expressed on chromosome 4, and its activation is associated with the progression of various malignancies. Somatic D816V mutation in Exon 17 of KIT is a significant driver and hallmark of SM [28]. Multilineage mutation of KIT D816V is associated with disease progression and advanced SM. KIT D816V mutation (c.2447 A>T, p.D816V) results in the substitution of aspartase to valine at codon 816 in kinase activation in the lead domain, resulting in a conformational change in the receptor [9]. This conformational change in the receptor results in ligand-independent constitutive activation of KIT and leads to increased cell proliferation and accumulation in various organs and reduced cell death [28]. The presence of multiple mutations in SM is associated with disease progression, drug resistance, and poor survival [29]. KIT D816V usually occurs as a late event in MC [30]. The presence of detectable KIT mutations is not universal in mastocytosis cases, and the lack thereof does not exclude the possibility of a mastocytosis diagnosis. A study looking at the molecular profiling of 19 patients with SM demonstrated the absence of a KIT mutation in 5%–10% of patients [29]. Possible reasons for not detecting a KIT mutation include false negatives due to low-sensitivity assays in the context of low MC counts, the existence of uncommon KIT mutations in different exons and codons that escape detection by standard KIT assays, and the potential inadequacy of myeloid gene panels in capturing KIT mutations, especially at low variant allele frequencies [31]. Using Sanger sequencing, our case was analyzed for possible mutations in KIT Exons 8, 9, 11, 13, and 17. He had an abnormal male karyotype with biclonal deletion of the chromosomes 7 and 11, ASXL1, and PTPN11 mutation. No alteration in FLT3, IDH1, IDH2, NPM1, and CBFB.

Currently, two proposed theories for the pathogenesis of SM are associated with AHN. The theories include activating C-KIT mutation with other genetic mutations in myeloid stem cells or transforming a subclone of myeloid progenitor cells through acquired C-KIT mutation [32]. This results in the proliferative advantage of mutated stem cells, leading to MC differentiation and proliferation [32]. In addition to C-KIT negative status, the patient's serum tryptase of 12.3 *μ*g/L was below the specified value of 20. However, a 2017 retrospective study exploring disease spectrum in patients with mastocytosis describes that a value greater than 11.4 *μ*g/L is considered elevated in most laboratories [33]. The serum tryptase criterion does not apply to patients with an associated clonal hematologic non-MC lineage disorder (AHN) since tryptase in such patients can originate from myeloid precursor cells [34].

Recent advancements in treating SM-AHN have focused on KIT-targeting tyrosine kinase inhibitors (TKIs), particularly midostaurin and avapritinib [35]. Midostaurin is a multikinase inhibitor with activity against both wild-type and D816V-mutated KIT, while avapritinib is a highly selective KIT D816V inhibitor. The National Comprehensive Cancer Network (NCCN) guidelines recommend avapritinib as a preferred treatment option for symptomatic SM, including SM-AHN [36]. These drugs are approved for KIT-positive SM-AHN, but their efficacy in KIT-negative cases remains unclear.

## 4. Conclusion

As this case shows, SM is rare and can manifest in various ways. The case presented several atypical features, such as the lack of dermatologic manifestations, KIT negativity, the lack of elevated tryptase, and the presence of SM-AHN. These all present diagnostic and therapeutic challenges. There have been recent advancements in treatment, but there is still a gap in the literature when dealing with nuanced cases. Future research should be aimed at elucidating the molecular landscape of KIT-negative SM to uncover novel genetic or epigenetic drivers. Collaborative efforts between clinical and research domains are essential to develop targeted therapies for this rare subgroup.

## Data Availability

Data sharing not applicable to this article as no datasets were generated or analyzed during the current study.

## Nomenclature

SM: systemic mastocytosis
AML: acute myeloid leukemia
SM-AHN: systemic mastocytosis with associated hematologic neoplasm
MC: mast cells
CM: cutaneous mastocytosis
ISM: indolent systemic mastocytosis
SSM: smoldering systemic mastocytosis
ASM: aggressive SM
MCL: mast cell leukemia
WBC: white blood cell
CD: cluster of differentiation
H&E: hematoxylin and eosin
IHC: immunohistochemistry
IgE: Immunoglobulin E
FISH: fluorescence in situ hybridization
SCF: stem cell factor
H&E: hematoxylin and eosin
IHC: immunohistochemistry
FISH: fluorescence in situ hybridization
TNF: tumor necrosis factor
SM-MPN: systemic mastocytosis with associated myeloproliferative neoplasm
SM-CMML: systemic mastocytosis with associated chronic myelomonocytic leukemia
JAK2: janus kinase 2
MDS/MPN: myelodysplastic/myeloproliferative neoplasms
ASM: aggressive systemic mastocytosis
